# In-situ synchrotron quantitative analysis of competitive adsorption tendency of human serum proteins on polyether sulfone clinical hemodialysis membrane

**DOI:** 10.1038/s41598-023-27596-2

**Published:** 2023-01-30

**Authors:** Amira Abdelrasoul, Ning Zhu, Huu Doan, Ahmed Shoker

**Affiliations:** 1 Department of Chemical and Biological Engineering, University of Saskatchewan, 57 Campus Drive, Saskatoon, SK S7N 5A9 Canada; 2 Division of Biomedical Engineering, University of Saskatchewan, 57 Campus Drive, Saskatoon, SK S7N 5A9 Canada; 3 Canadian Light Source, 44 Innovation Blvd, Saskatoon, SK S7N 2V3 Canada; 4 Department of Chemical Engineering, Toronto Metropolitan University, 350 Victoria St, Toronto, ON M5B 2K3 Canada; 5 Nephrology Division, College of Medicine, University of Saskatchewan, 107 Wiggins Rd, Saskatoon, SK S7N 5E5 Canada; 6 Saskatchewan Transplant Program, St. Paul’s Hospital, 1702 20Th Street West, Saskatoon, SK S7M 0Z9 Canada

**Keywords:** Biochemistry, Biotechnology, Chemical biology, Health care, Chemistry, Engineering, Materials science, Mathematics and computing

## Abstract

Comprehensive understanding of protein adsorption phenomenon on membrane surface during hemodialysis (HD) is one of the key moments for development of hemocompatible HD membrane. Though many mechanisms and kinetics of protein adsorption on some surface have been studied, we are still far away from complete understanding and control of this process, which results in a series of biochemical reactions that causes severe complications with health and even the death among HD patients. The aim of this study is to conduct quantitative analysis of competitive adsorption tendency of human serum protein on polyether sulfone (PES) clinical dialysis membrane. In situ synchrotron radiation micro-computed tomography (SR-µCT) imaging available at the Canadian Light Source (CLS) was conducted to assess human serum proteinbinding and undertake the corresponding quantitative analysis.The competitive adsorption of Human protein albumin (HSA), fibrinogen (FB) and transferrin (TRF) were tested from single and multiple protein solution. Furthermore, *in-vitro* human serum protein adsorption on clinical dialyzers was investigated using UV–Visible to confirm the competitive adsorption tendency. Results showed that when proteins were adsorbed from their mixture, FB content (among proteins) in the adsorbed layer increased from 3.6% mass (content in the initial solution) to 18% mass and 12%, in case of in situ quantitative and *invitro* analysis, respectively. The increase in FB content was accompanied by the decrease in the HSA content, while TRF remained on approximately on the same level for both cases. Overall, the percentage of HSA adsorption ratio onto the HD membrane has dropped approximately 10 times when HSA was adsorbed in competition with other proteins, compared to the adsorption from single HSA solution. The substitution of HSA with FB was especially noticeable when HSA adsorption from its single solution was compared with the case of the protein mixture. Moreover, SR-µCT has revealed that FB when adsorbed from a protein mixture solution is located predominately in the middle of the membrane, whereas the peak of the distribution is shifted to membrane bottom layers when adsorption from FB single solution takes place. Results showed that HSA FB and TRF adsorption behavior observations are similar on both *in-situ* small scale and clinical dialyzer of the PES membrane.

## Introduction

Hemodialysis is an essential blood cleansing technique that is currently required for more than 2 million patients worldwide to remove metabolic waste products, toxins, salts and extra water that accumulate in patients with end-stage kidney (ESKD) and other diseases^1^. The major functional part of a hemodialysis system is a polymeric membrane that separates the undesirable components from the blood plasma.

Polymeric materials are widely used in many industrial applications, including membrane technology. Among various polymers used for membrane fabrication, polyether sulfone (PES) is one of the most popular polymers for membrane fabrication due to its excellent mechanical properties, thermal and chemical stability, and resistance to swell in water. Thus, PES membranes have been successfully applied in many commercial and industrial applications, including wastewater treatment, gas separation, biomedical applications and many others for more than 30 years^2–6^. Combination of PES properties and its commercial availability resulted in PES being used in more than 90% of the dialyzers in the world^7^.

Many studies have demonstrated that we can synthesize biocompatible HD membrane, however without knowing precisely how each protein interacts with the membrane material and how can we determine which protein should adsorb first and trigger blood activation. The adsorption of each protein leads to different pathway of blood activations and consequently different patient outcomes, in addition the adsorption of proteins block HD membrane pores and significantly reduce the toxins clearance efficiency.When blood comes into contact with foreign bodies like hemodialysis membrane, serum proteins are adsorbed onto the membrane surface, which results in a sequence of coagulation, complement activation and coagulation processes, leading to further health problems and being responsible for increased mortality^8–11^. Although many researchers have devoted to study of mechanism and kinetics of protein adsorption onto various surfaces^12–15^, membrane fouling during hemodialysis is still not well understood. Our research group focused on a study of hemodialysis (HD) polymer membranes that were used in actual clinical applications^16–18^. We were able to enhance the PES HD membrane’s performance by controlling fiber diameter and surface morphology, as well as improve membrane antifouling properties by surface modification with zwitterionic coatings^16,17,19–21^. We also demonstrated how fibrinogen (FB) adsorption and hydrodynamic conditions influence complement activation, inflammatory and thrombotic responses^18^. Moreover, the influence of membrane morphology and hydrophilic properties on the membrane interaction with proteins and inflammatory biomarkers was studied^21,22^. Also, from docking studies, we have demonstrated that sulfone functional groups in PES played an important role in interacting with human serum proteins and other biological molecules^7^. However, understanding the protein adsorption behavior within the hemodialysis system is a crucial step for the development of a controlled hemodialysis process without severe consequences for patients’ health. In addition, developing membrane with less interactions with human serum protein would enhance the biocompatibility of hemodialysis membranes and lead to the development of membrane materials that promote attenuated blood activation reactions.

Synchrotron-based imaging is a powerful tool which allows the 3D real-time visualization of the protein deposition without interfering in the ultrafiltration process. Therefore, the present study was aimed to gain in-depth understanding of competitive adsorption tendency of human serum proteins (HSP) on polyether sulfone (PES) clinical dialysis membrane, which is commonly used in hospitals worldwide. The objectives of the study were to: (i) investigate HSP adsorption mechanism from single and multi-protein solutions onto the PES membrane using novel *in-situ* synchrotron imaging and corresponding; (ii) obtain quantitative analysis of HSA, FB, TRF adsorption inside the membrane matrices; and (iii) experimentally asses and validate the adsorption of HSP from multiprotein HSP solution using PES clinical dialyzer.

## Competitive human serum protein adsorption and vroman effect

Each human serum protein has different interaction affinity with HD membrane surface, which triggers a different pathway of blood activation cascades. As proteins are adsorbed to the surface, the composition of the protein layer is gradually changing, dependent on the interactions and the repulsion forces between proteins, followed by the cake build-up, which significantly affects the ultrafiltration process of blood and uremic toxins clearance efficiency.

**Figure 1:**
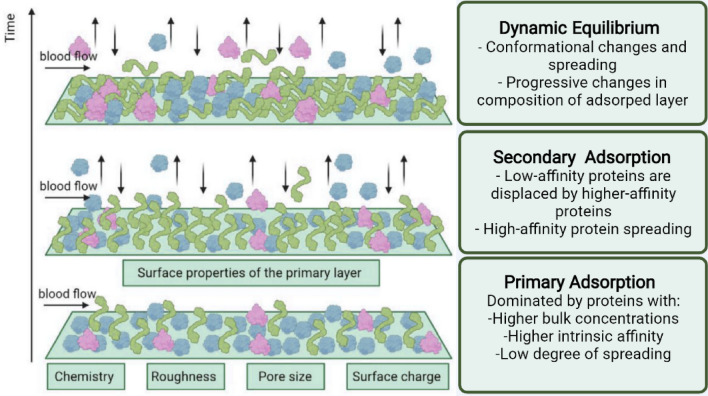
Illustration of competitive human serum protein adsorption and Vroman effect on dialysis membrane.

In our recent study, our research group has proposed that multiprotein adsorption dynamics on HD membrane, to be occurred in three consequent stages, primary adsorption, secondary adsorption and dynamic equilibrium, as presented in Fig. 1. Table 1 summarizes the structure description and size of human serum albumin, fibrinogen, and transferrin^23^.

**Table 1:** Structure Description and Size of Major Human Serum Proteins.

Protein	Structure description	Size
Albumin	Globular protein, single peptide chain protein, 585 amino acid, three homologous domains (I, II, and III)	67 kDa
Fibrinogen	Glycoprotein, 2 identical monomers, 3 non-identical peptide chains (α, β, and γ), trinodal structure	342 kDa
Transferrin	Monomeric glycoprotein, two homologous lobes (N- and C-lobes) connected by a short peptide	80 kDa

Smaller proteins adsorb first due to higher diffusivity and ability to penetrate deeper into the membrane pores and as the first layer on the membrane surface; and hence they are the predominant species in the primary adsorption stage, presented in Fig. 1. In addition, the multiprotein adsorption process is dominated by proteins with higher concentration, higher intrinsic affinity, and low degree of spreading^23^. Furthermore, this stage is controlled by the chemistry, surface roughness, pore size and surface charge of HD membrane. On the other hand, larger proteins would diffuse at a slower rate towards the membrane surface. Nevertheless, due to their larger surface area with more active sites, they tend to bind more strongly to the surface.

At the second stage of adsorption, low-affinity proteins are displaced by higher-affinity proteins, and high-affinity protein spreading, as presented in Fig. 1. The adsorption of proteins to the membrane surface takes place within milliseconds, but the spreading of the proteins is a slower process than can take several hours^24–26^.

Following this stage, the interaction between protein–protein and protein-membrane induced a gain of free energy so the protein molecule undergoes conformational changes and spread on the membrane surface, as a part of the third stage. Depending on the degree of interaction, a large protein can dislocate a small one in a phenomenon called the Vroman effect. This dynamic interaction and equilibrium, presented in Fig. 1, lead to progressive changes in the composition of the protein cake layer, affecting the filtration performance and biocompatibility profile of the surface^27–31^.

## Materials and methods

### Materials

PES clinical membrane modules utilized in Canadian hospitals were used in our study. The membrane material is based on blended polymer of polyaryl ethersulfone polyvinylpyrrolidone (PAES-PVP) (or PAES) (REVACLEAR 400 dialyzer). These medical-grade membranes were provided by St Paul’s Hospital, Saskatoon, Canada. Membrane total active surface area was1.4m^2^. Human serum proteins (HSA, FB and TRF) and phosphate buffer solution (PBS) were purchased from Sigma-Aldrich. Gold nanoparticles were purchased from Nanopartz™. These nanoparticles were conjugated to human proteins (albumin, fibrinogen and transferrin) to be visualized in the SR-μCT. Saline and dialysate solutions were obtained from Baxter.

### Research methods

#### In situ synchrotron advanced imaging techniques at BioMedical Imaging and Therapy (BMIT) Beamline

**Figure 2:**
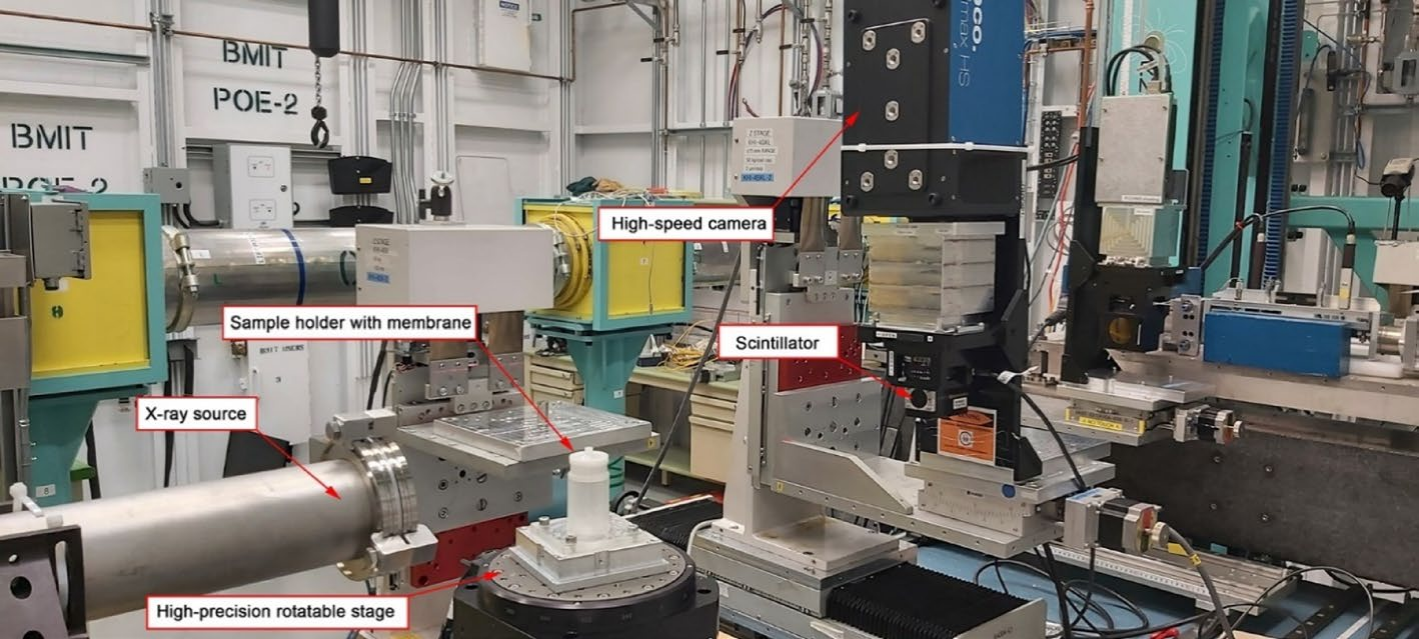
Photo of the experimental SR-μCT system in BMIT hatch at the Canadian Light Source (CLS).

Visualization of protein adsorption was proceeded using a monochromatic beam at 20 keV energy. A beam monitor AA-40 (500 μm LuAG scintillator, Hamamatsu, Japan) coupled with a high-resolution camera PCO Dimax HS (PCO, Germany), providing a pixel size of 5.5 μm and a field of view (FOV) of 4.4 mm × 2.2 mm, was used to record the X-ray radiographs. The high photon flux allowed very detailed observation of particle deposition in microscopic layers of the membrane. CT projections were recorded at 30 keV at the 05ID-2 beamline of the BioMedical Imaging and Therapy (BMIT), at the Canadian Light Source (CLS). A photo of the experimental setup is shown in the Fig. 2.

The obtained radiographs were converted into graphical images using the Avizo software. Further image analysis was performed using the image J software. Gold nanoparticles conjugated with proteins produced brightest spots on the image, thus providing the quantitative information about the protein amount, adsorbed at each scanned layer. In case of adsorption from multiprotein solution, each protein was detected and analyzed on the basis of specific shape of nanoparticles used for conjugation with each protein. Thus, spherical particles were used for conjugation with HSA, rods (sphericity ratio of 0.85) for FB and cylinders (sphericity ratio of 0.91) for TRF. The Avizo software was used to convert the images to quantitative analyses. Membrane thickness was modeled by 7 Regions of interest (ROI). Region 1 represents the very top membrane surface. The bottom membrane parts are located in Region 7. In order to ensure the accuracy of the data, four measurements were carried out for each sample at different spots. The presented data in the discussion is an average of the measurements. Each protein was highlighted in a different color in Avizo images, i.e. HSA is colored with green, FB—purple and TRF—cyan.

#### In-vitro human serum protein adsorption investigation using UV–visible

*In-vitro* human serum protein adsorption was conducted and validated on a clinical PES dialyzer using the multiprotein mixture that simulated the patient’s blood at a flow rate of 200 ml/min and a dialysate flow of 500 ml/min. The simulated protein solution was made of albumin, fibrinogen, transferrin or their mixture from human plasma (Sigma-Aldrich), saline (0.9% NaCl Injection USP, Baxter) and phosphate buffer solution (1.0 M, pH 7.4 at 25 °C, Sigma-Aldrich). The concentration of proteins simulated the average concentration of the proteins in male and female bodies, i.e. HSA, FB, TRF had the concentration of 50 mg/mL, 2 mg/mL, and 3 mg/mL, respectively. Samples of known concentration of proteins for UV–visible calibration were carefully prepared at room temperature (22 °C) using NaCl, 0.9% saline solution (Baxter) and proteins from human plasma. The pH of the solution was adjusted to 7.2 using PBS. The samples were analyzed using a UV–vis spectrometer (Flame, Ocean Optics). The UV/Vis spectra for known concentrations of proteins for the calibration are presented in Fig. S.1.

#### Scanning electron microscopy

Scanning procedure was performed using the Hitachi SU8010 device. Images were collected at 3 kV acceleration voltage. For all membranes, care was taken to avoid burring up the gold (Au)-precoated (10 nm; Quorum Q150T ES) samples while collecting images at high magnifications.

### Ethical approval

The principal investigator of the project, Dr. Amira Abdelrasoul, has the Research Ethics Approval and the Operational Approval to conduct the research in Saskatchewan Health Authority, in Canada. She has the responsibility for the regulatory approvals that pertained to this project, and for ensuring that the authorized project was conducted according to the governing law. All the experimental protocol for involving humans was conducted according to the governing law. All the participants in this study, from the hemodialysis center at St. Paul Hospital, have signed the written informed consent, approved by the Biomedical Research Ethics Board (Bio-REB).

## Results and discussion

### PES hemodialysis membrane morphology

**Figure 3:**
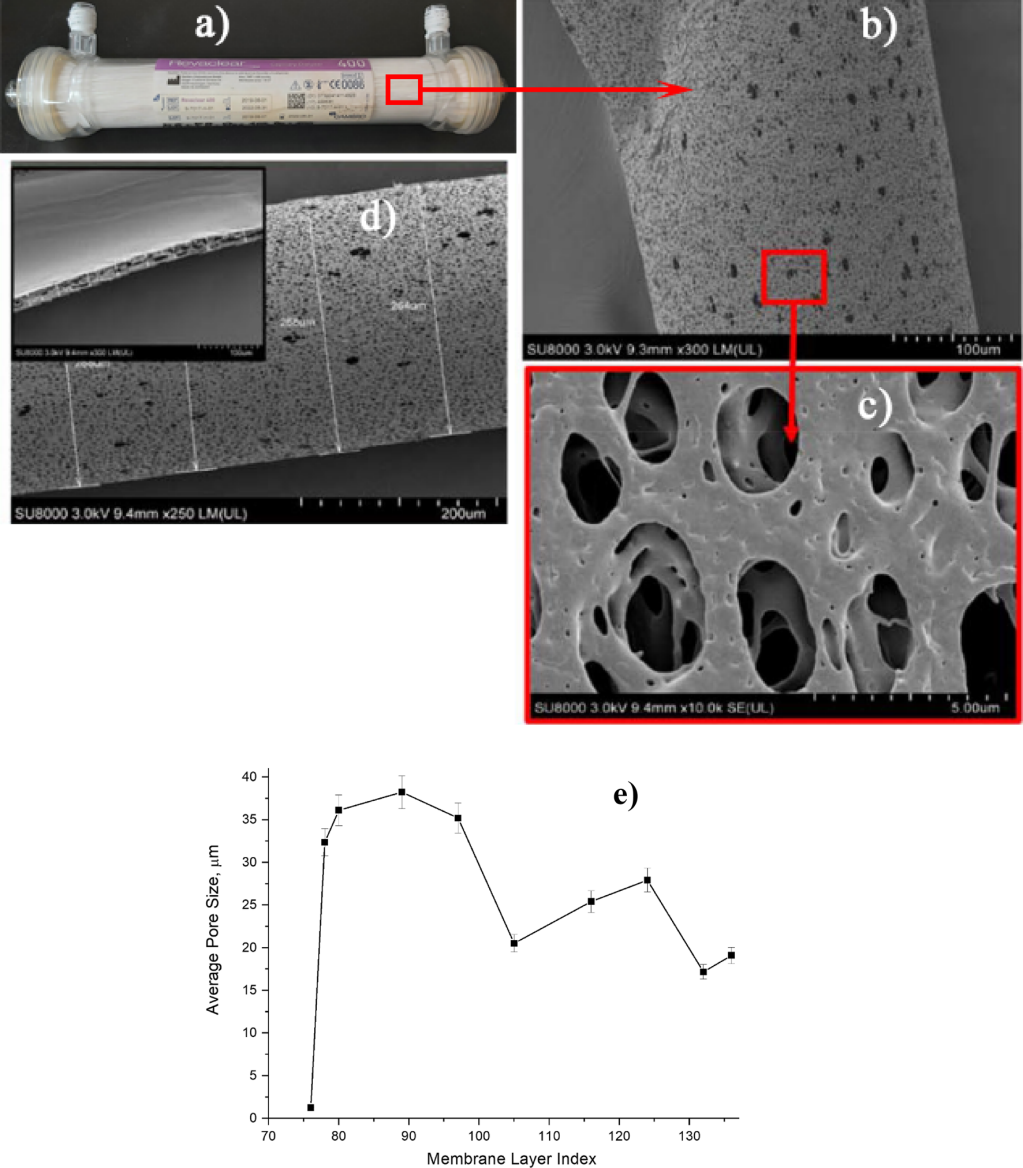
(**a**) PES hollow-fiber based membrane module; (**b**–**d**) SEM images of PES hollow fibers; and (**e**) average pore size of PES sheet membrane across thickness, using SR-μCT.

PES membrane module (Fig. 3a) consisted of hollow fibers with the structure presented in Fig. 3b,c. The hollow fibers have 275 μm external diameter with walls thickness about 25 μm and pores diameter around 2-4 μm. As estimated by SR-μCT analysis (see Fig. 3d), the pore sizeincreased from about 2.5 to 38 μm with the increase of layer index. However, the pore size decreased from 38 to 18 μm when further moving from the membrane fiber-containing surface toward to the porous film layer, i.e. further increases in the layer index (see Fig. 3e).

### In situ* investigation on protein adsorption from single and multi-protein solutions*

#### Adsorption from protein mixture

**Figure 4:**
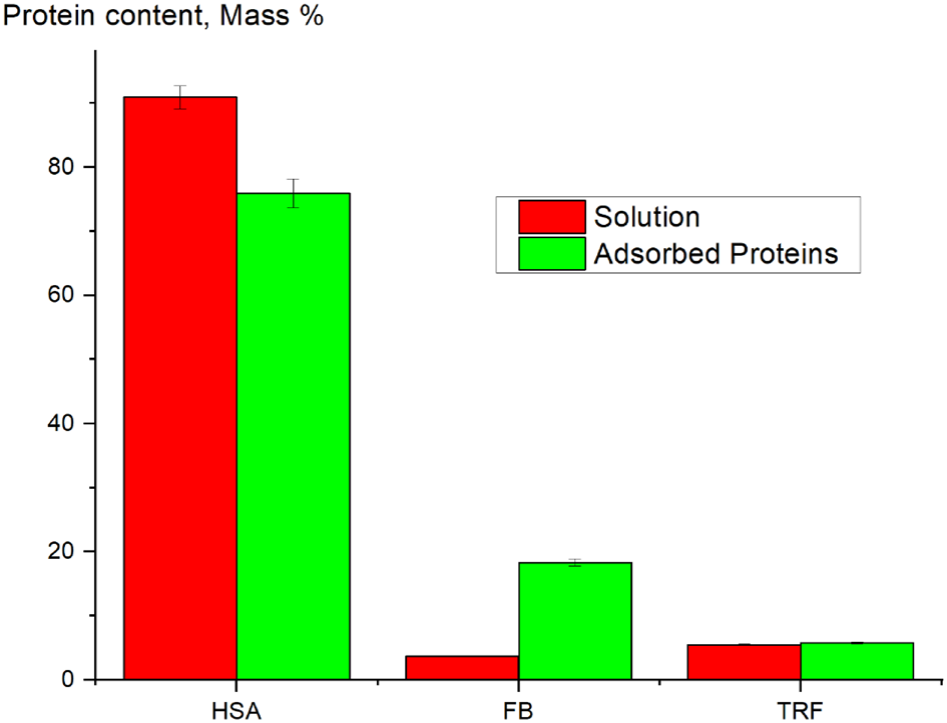
Comparison between the protein composition in the initial solution and that in adsorbed proteins from a multi-protein solution.

Protein adsorption from a protein mixture, containing 50 mg/mL, 2 mg/mL, and 3 mg/mL of HSA, FB, TRF, respectively, was investigated using synchrotron SR-μCT analysis. This analysis can be used for reconstruction of protein distribution within both external and internal membrane structure in each membrane layer, as well as obtaining information about the total amounts of adsorbed proteins. In the current research it was expected that the composition of proteins adsorbed on the membrane was different from the protein composition of the initial multi-protein solution (see Fig. 4). HSA content in the adsorbed protein decreased, while an increase in the FB content was detected. HSA content in the initial multi-protein solution was about 91%, whereas adsorbed proteins contained only 76% of HSA. This change in HSA amount was compensated by an increase in the FB content. It should be pointed out that the FB content in adsorbed protein increased drastically from 4% in the initial solution to 18% in the adsorbed protein after 1 min of treatment. This is in agreement with our previous work^22^. Noticeably, the TRF content in the adsorbed protein mixture was about the same as that in the initial solution.

**Figure 5:**
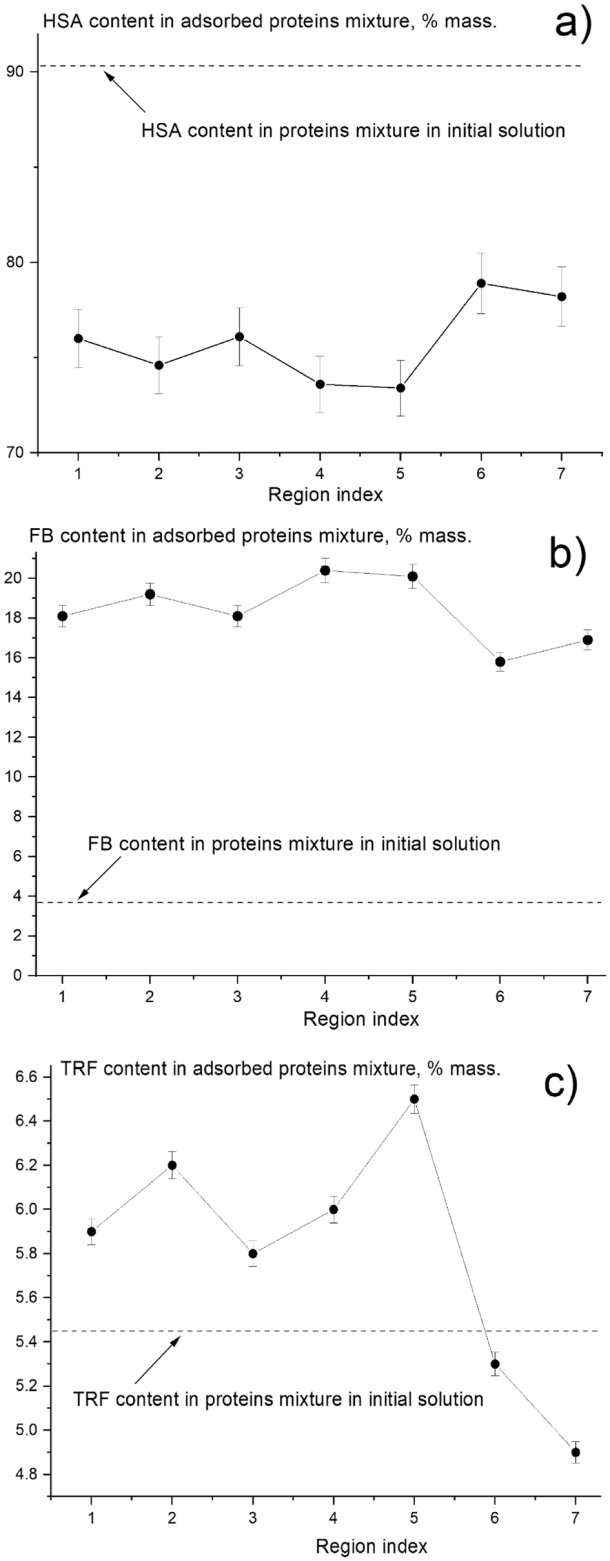
Protein content in the adsorbed protein mixture in each membrane region index: (**a**) HSA; (**b**) FB; (**c**) TRF.

Figure 5 presents the change in the composition of the adsorbed proteins in each membrane region index compared to the protein content in the initial solution. For instance, Fig. 5a presents % of HSA adsorbed at each layer, which was calculated as HSA adsorbed mass divided by the total mass of adsorbed mixture of proteins at each layer (HSA + FB + TRF); and compared to the HSA% in the initial solution. The concentration of a protein in the adsorbed layer, based on the total amount of all proteins at that layer, is dependent on protein affinity with HD membrane, the competition with other protein for adsorption sites, and diffusivity besides its concentration in the liquid mixture.

When considering the protein distribution across the membrane thickness, one can see that the maximum content of FB and TRF (Fig. 5b,c, respectively), and the least content of HSA (Fig. 5a) are located in the first 5 membrane regions. Our multiprotein adsorption dynamic proposed that smaller proteins would be adsorbed first due to their higher diffusivities, and hence, they would be the predominant species in the primary adsorption stage, as presented in Fig. 1. According to Table 1, HSA, FB and TRF have a molecular weight of 67 kDa, 342 kDa, 80 kDa, respectively. Therefore, TRF potentially could penetrate deeper into the membrane (larger region index) as compared to FB and comparable to HSA. Nevertheless, the concentration of TRF was about 5.4% of the initial mixture solution compared to the HSA, which was about 90% of the initial solution. Consequently, most of TRF might have been already adsorbed in the initial regions, resulting in much lower adsorbed amounts of TRF in regions 6 and 7 (5.3% and 4.9% respectively), as presented in Fig. 5c. The maximum TRF content reached 6.5% at the 5^th^ membrane region. Also, the TRF content distribution was much less uniform, as compared with those of HSA and FB.

On the other hand, HD membrane got enriched with HSA. The concentration of HSA was very high in the initial solution (90%), and HSA was the smallest molecular weight of 67 kDa. Thus, HSA could penetrate into the membrane the most, resulting in a higher amount of HSA adsorbed at the deeper regions (6 and 7), as presented in Fig. 5a. It is worth noting that among the three proteins, the FB illustrated the highest adsorption affinity to the membrane surface. The amount of FB adsorbed to the membrane, relative to the other proteins, was much higher than its concentration in the solution (see Fig. 5b). This might have help sustaining adsorption of FB throughout the membrane thickness even though the concentration of FB in the solution was the lowest (less than 4%) and the molecular weight was the highest.

**Figure 6:**
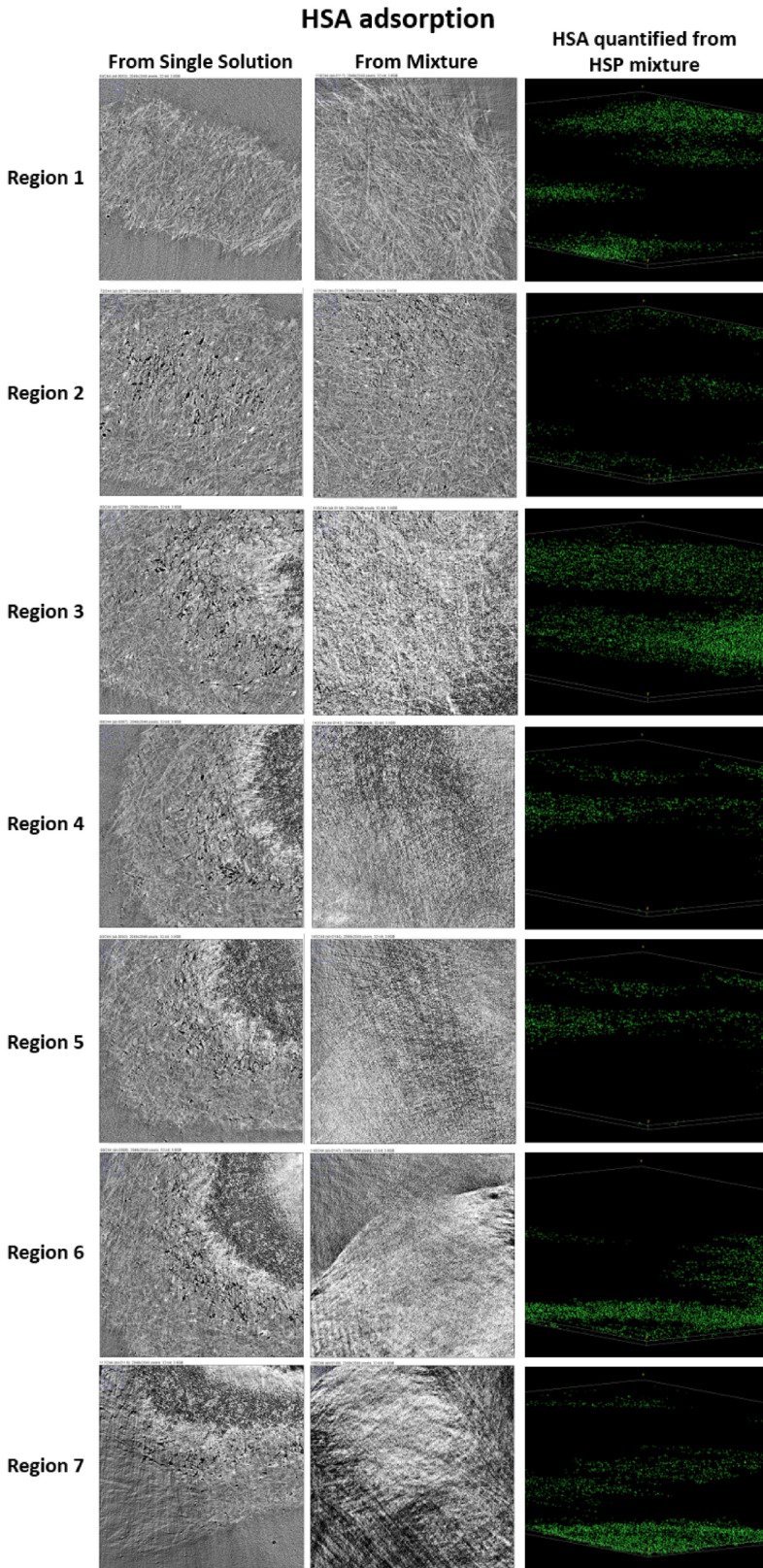
Converted radiographs of the PES membrane with adsorbed HSA from its single solution and HSP, and visualization of the HSA distribution across the membrane thickness.

**Figure 7:**
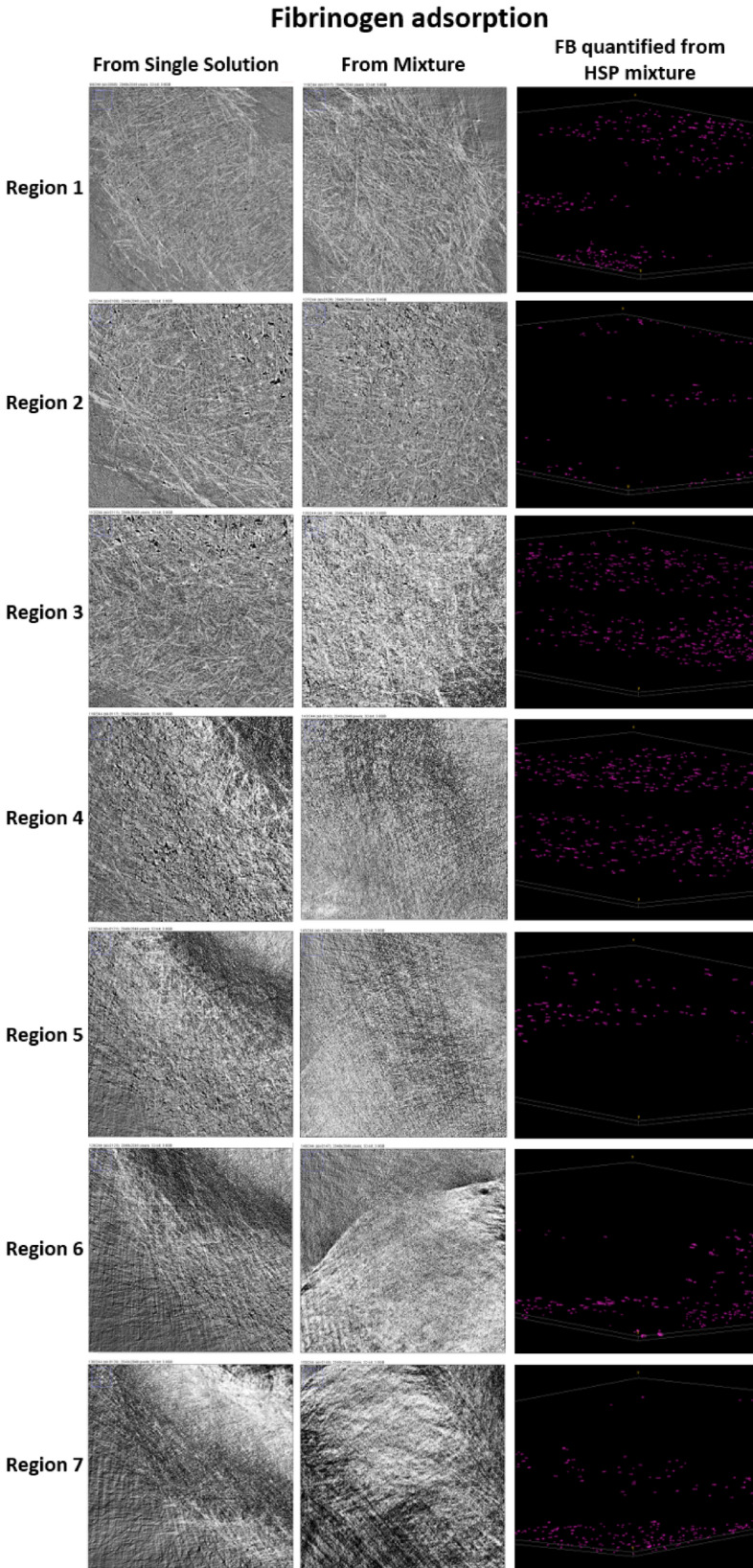
Converted radiographs of PES membrane with adsorbed FB from its single solution and HSP, and the visualization of the FB distribution across the membrane thickness.

**Figure8:**
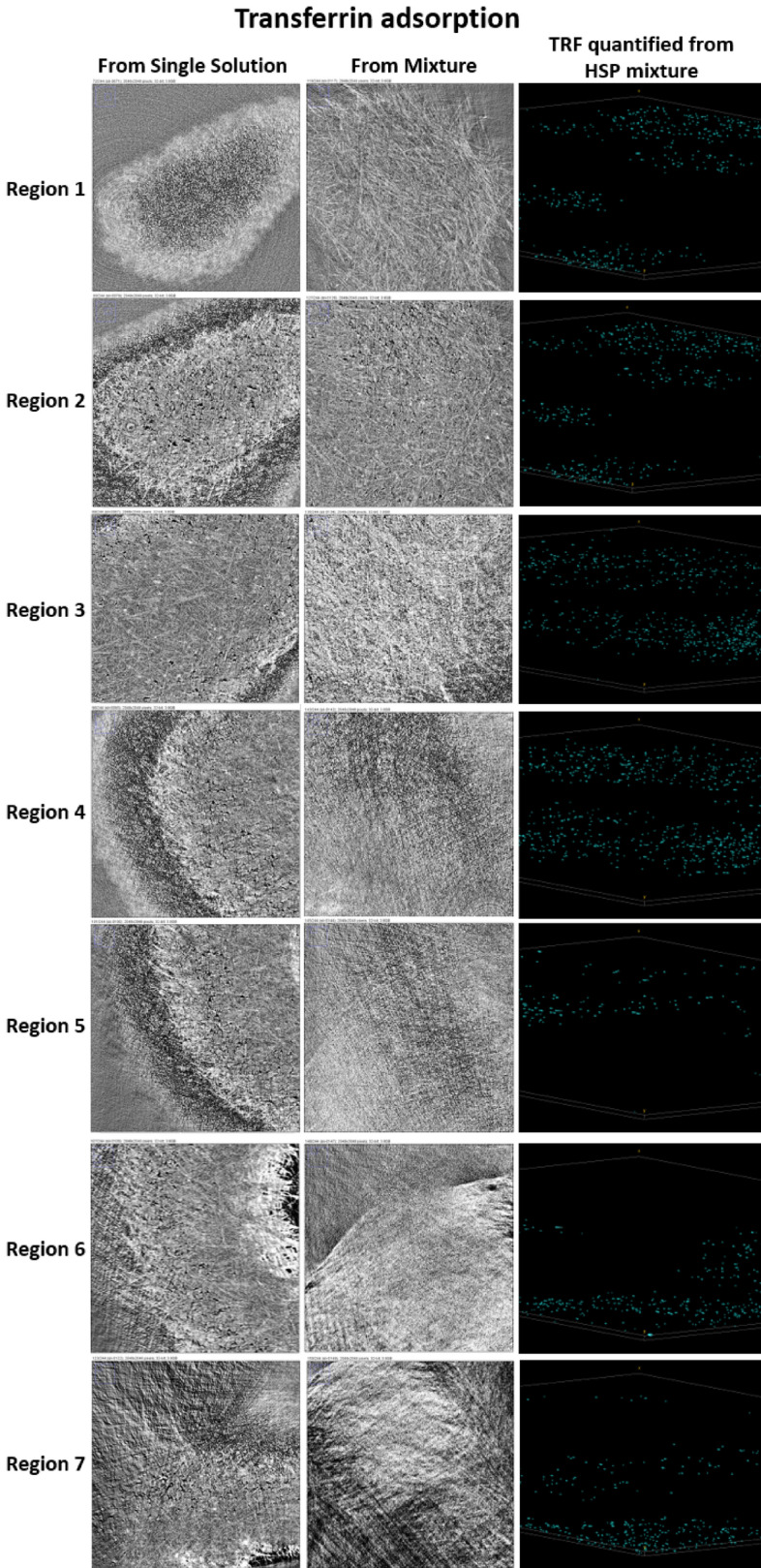
Converted radiographs of PES membrane with adsorbed FB from its single solution and HSP, and visualization of the FB distribution across the membrane thickness.

Based on the difference in the shapes of gold nanoparticles used for the conjugation with each protein, it was possible to reconstruct each protein’s location even in the case of adsorption of the human serum protein mixture (HSP). The radiographs, converted to graphical images and the distribution of each protein across the membrane thickness are given in Figs. 6, 7 and 8. Then, a quantitative analysis of the images was carried out using the Avizo software.

#### Adsorption from single-protein solutions

**Figure 9:**
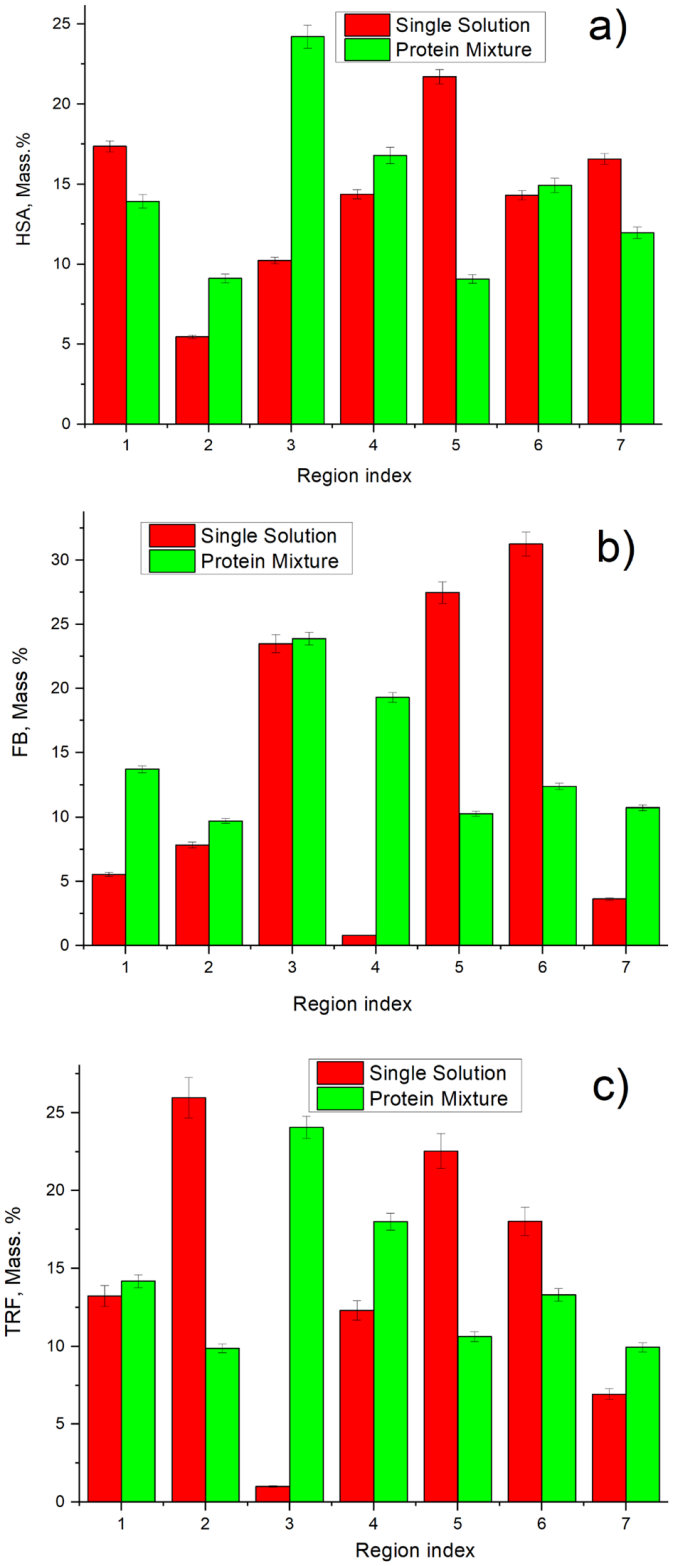
Protein distribution across the membrane thickness when adsorbed from single solution and from proteins mixture: (**a**) HSA; (**b**) FB; (**c**) TRF.

Besides adsorption from the protein mixture, adsorption from single-protein solutions was also studied. Figure 9 presents the comparison of each protein distribution across the membrane thickness for adsorption from single-protein and multi-protein solutions. For instance, Fig. 9a presents %HSA adsorbed at each layer with respect to the total amount of HSA adsorbed across all regions over the whole membrane thickness, which is equal to the mass of HSA adsorbed at a given region divided by the total mass HSA adsorbed (HSA adsorbed at the 1st region + HSA adsorbed at the 2nd region + … + HSA adsorbed at the 7th region) and converted to percentage.

HSA (Fig. 9a) appeared to be distributed uniformly across the membrane thickness, though some local increase in the protein content was observed in the middle regions for both single and multi-protein solution adsorption. FB (Fig. 9b) was distributed less uniformly and there was some difference between single-protein and protein mixture adsorption. FB tended to locate predominately in the center of the membrane when it was adsorbed from the protein mixture solution, whereas the higher content region was shifted towards the membrane bottom film layer for adsorption from the single-protein solution. It is worth noting that adsorption of FB on a HD membrane surface is highly undesirable process, which is considered as the first step for further platelet adhesion, their activation and further triggering of biochemical cascade reactions, causing blood clotting and severe health problems for HD patients^18^. TRF’s distribution (Fig. 9c) was very similar to that of FB. Nevertheless, at region 3, the adsorbed TRF from the single-protein solution was very small compared to those in other regions of the membrane. Only the adsorbed amount for the multi-protein mixture was higher in the central region.

**Figure 10:**
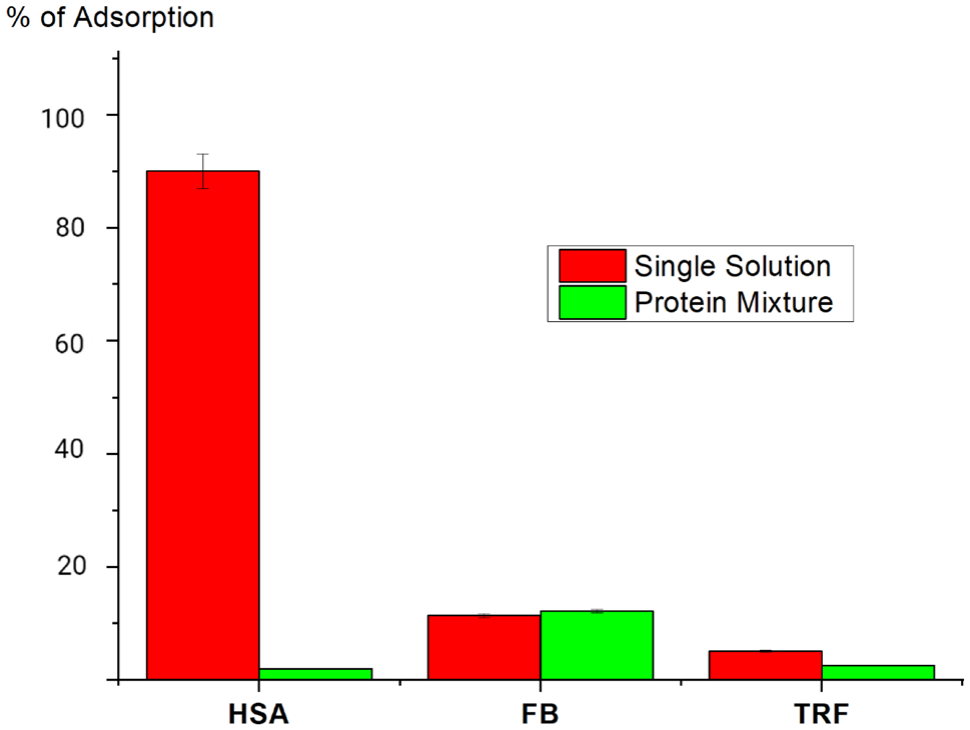
Comparison of adsorption ratio* of each protein from its single solution and protein mixture (* the mass of adsorbed protein divided by the total mass of all protein adsorbed × 100).

Despite of the similarity in the protein distribution, there was a significant difference in the relative amount of protein adsorbed (see Fig. 10). The percentage of FB adsorption, was almost independent from the type of the solution, i.e. a single-protein solution or a multi-protein solution. For TRF, the adsorbed amount for the single-protein solution was about twice that with the protein mixture. Moreover, the percentage of TRF adsorbed was about a half of that for FB. The significant difference in the relative adsorbed amount was observed with the HSA protein. The percentage of HSA adsorbed from single protein solution was about 10 times higher than that of the HSA absorbed from the protein mixture (see Fig. 10). The main reason of this behavior is believed to be the presence of FB in the adsorbed layer.

As it was demonstrated before (see Fig. 5, for % in each membrane layer), when the membrane was used with the protein mixture, the composition of adsorbed proteins differed significantly from the protein composition in the initial solution, i.e. the FB content increased from 4% (in the initial solution) to 18% (in the absorbed layer), as shown in Fig. 5b, whereas the HSA amount decreased from 91% (HSA% in the initial solution) to 77% (in the absorbed layer), as presented in Fig. 5a. This indicates the preferential adsorption of FB over HSA; and hence, the amount of HSA adsorbed decreased drastically with the multi-protein solution compared with the case for the single-HSA solution.

### Influence of FB on competitive adsorption tendency of human serum proteins

It is worth mentioning that the mechanisms involved in blood protein adsorption to dialysis membrane is a complex phenomenon due to the highly heterogeneous composition of blood^23^. In overly simplified terms, first, the proteins approach the membrane via the diffusion mechanism. Then, protein molecules adhere to the membrane as a result of static interactions and finally, the protein molecule undergoes conformational changes at the membrane surface^23^. Therefore, the membrane surface charge plays a crucial role in those interactions. In our study, PES membrane has a high negative surface charge of − 68 mV.

**Figure 11:**
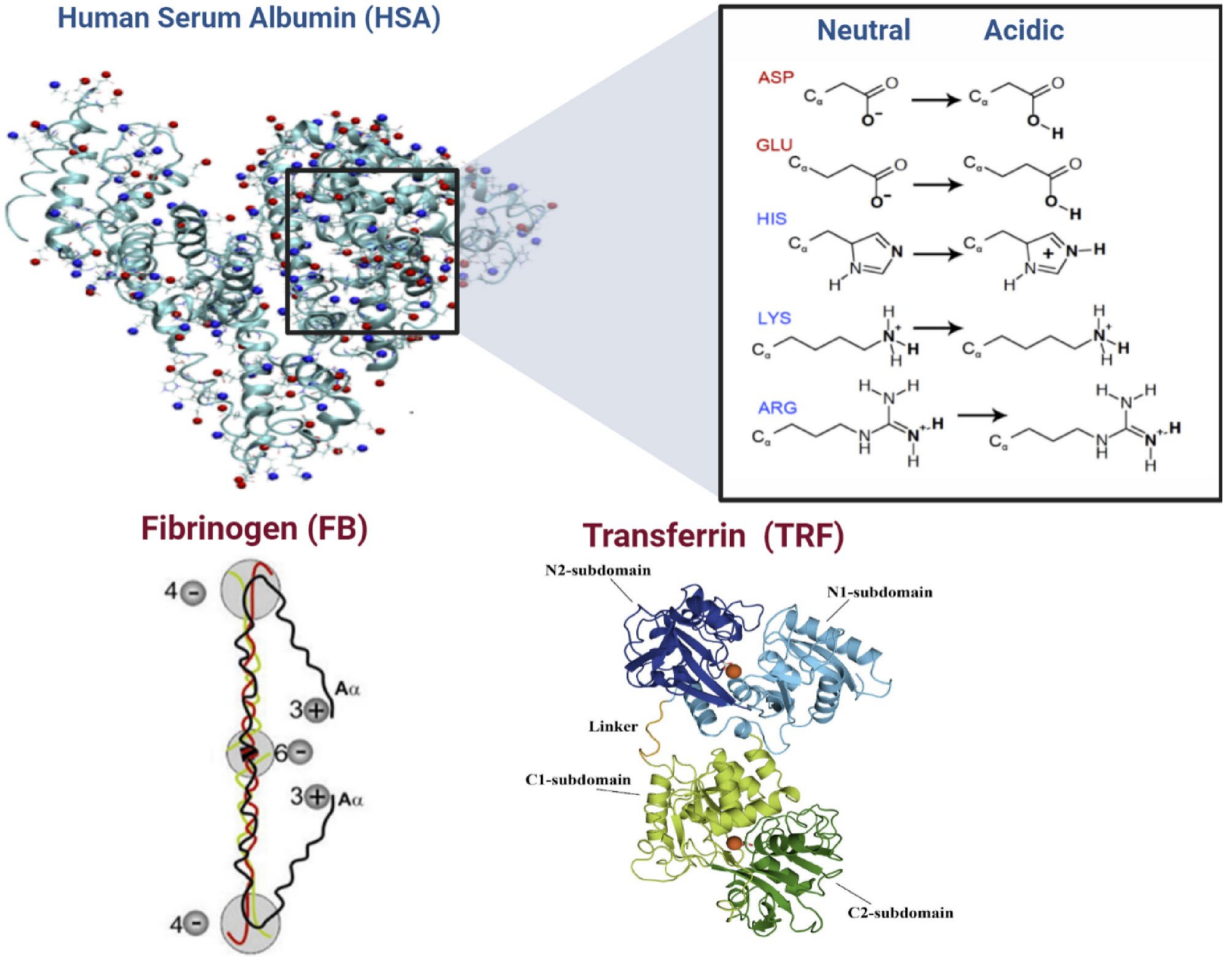
Human Serum Protein Structure and Charge.

A closer look at HSA and FB structure and charge reveals its tendency to interact and the mechanism of human protein adsorption. HSA structure exposes it “patchy and anisotropic” nature. Its heart-shaped structure is a result of a single polypeptide forming a 3-D structure composing of three similar domains, namely Domain I, II, and III, as mentioned in Table 1. HSA is comprised of hydrophobic and hydrophilic regions^32^. Nevertheless, it also possesses pockets/cavities with strong hydrophobic properties. Examples of such hydrophobic cavities are Sudlow site I and Sudlow site 2, which are in subdomains IIA and III A. Though the three domains of HSA are structurally comparable, they differ in terms of amino acid sequences. Percentagewise, the similarities between domains 1 and 2, domains 1 and 3, and domains 2 and 3 are 25%, 18%, and 20%, respectively. These differences among the domains result in non-homogenous distribution of hydrophobic patches and charges of HSA^32^. As presented in Fig. 11, HSA has residues of ASP, GLU, HIS, LYS, ARG. The HIS, LYS and ARG residues are hydrophobic, and only ASP and GlU residues are hydrophilic. Furthermore, in the acidic media of dialysis patients’ blood, all the residues would be hydrophobic as shown in Fig. 11.

On the other hand, fibrinogen (FB) molecular mass is 342 kDa, as mentioned in Table 1, and it consisted of 6 polypeptide chains (2 Aα, 2 Bβ, and 2γ). The Aα, Bβ, and γ polypeptide chains are made up of 610, 461, and 411 amino acid residues, respectively^33,34^. The chains are held together by disulfide bonds, and they intertwine forming a structure of globular regions which are connected. These regions are the E domain, two D domains and two αC domains. The E domain contains the N-terminals of the all the polypeptide chains, whereas the D domains combines the C-terminals of the Bβ, and γ chains. The remaining C-terminals of the Aα forms the two αC domains^35,36^. The E domain are linked to the two D domains by α-helical coiled coils. In comparison to the αC domains, the E and D domains are much more hydrophobic. Furthermore, at physiological pH, the αC domains are positively charged while the E and D domains possess negative charges. In addition, FB illustrated the anisotropic charge distribution of fibrinogen by computing the local charges of each domain^37^. For instance, at pH of 7.4, they determined the charges of the local charge of the E domain, D domains (2 domains), and α-C domains (2 domains) to −6, −4, and + 3, respectively, as shown in Fig. 11.

Therefore, HSA attaches intensively from single protein to the negative PES surface due to its hydrophobic nature due to electrostatic interactions. However, in the case of protein mixture, after a few HSA bind on PES surface, the FB would intensively compete with HSA because of its unique structure with anisotropic distribution charges. Hence, FB content increased from 4% (in the initial solution) to 18% (in the absorbed layer), as shown in Fig. 5b, whereas the HSA amount decreased from 91% (HSA% in the initial solution) to 77% (in the absorbed layer), as presented in Fig. 5a. This indicates the preferential adsorption of FB over HSA; and hence, the amount of HSA adsorbed decreased drastically with the multi-protein solution compared with the case for the single-HSA solution.

On the other hand, FB has a lower tenancy to compete with TRF adsorption. TRF is structured in two homologous lobes (N- and C-lobes) connected by a short peptide. C-lobe contains a carbohydrate moiety attached to it. Each lobe is divided into subdomains that connect two antiparallel β-sheets that act as flexible joints, as presented in Fig. 11. In our previous work, we used molecular docking to estimate the binding affinity of TRF to PES polymeric membrane material. We observed that TRF possesses a higher binding affinity of −7.9 kcal/mole compared to FB of −6.00 kcal/mole. This can be an indication that FB cannot significantly influence the adsorption of TRF molecules compared to HSA in a phenomenon described as the Vroman effect (Fig. 1).

### In-vitro human serum protein adsorption on clinical dialyzers using UV–visible

**Figure 12:**
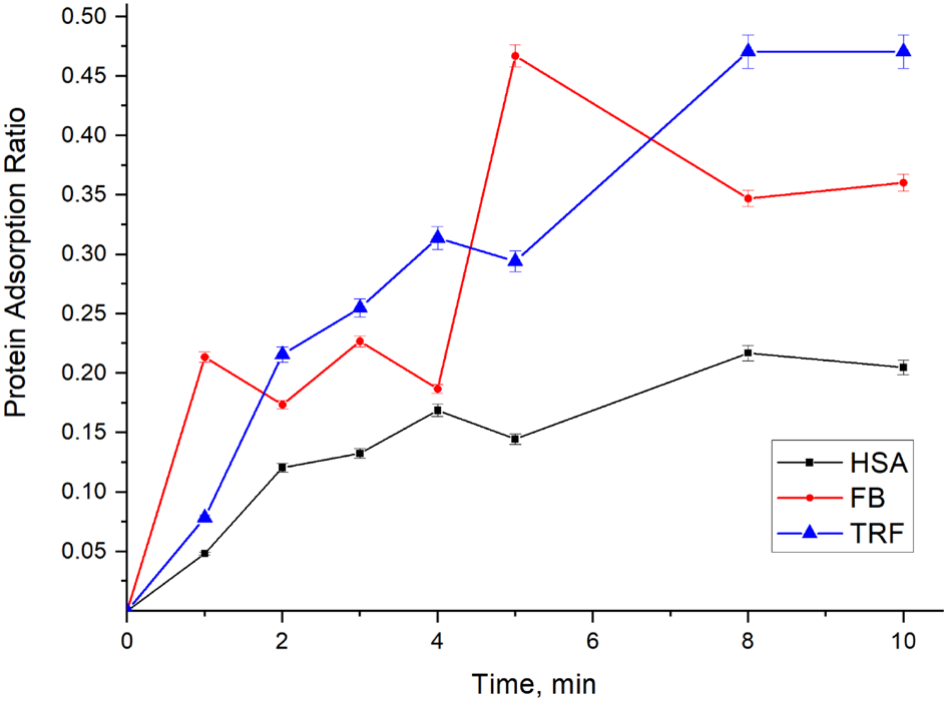
Proteins adsorption ratio* with time on PES clinical dialyzer (*adsorption ratio is the mass of adsorbed protein divided by the total mass of all protein adsorbed × 100).

Protein adsorption in PES membrane module was investigated by UV analysis of protein solution before and after ultrafiltration using PES clinical dialyzers. Based on the UV calibration curves recorded for known concentrations of single proteins and their mixtures, the concentration of each protein in the protein outlet stream of the protein (retentate) was measured; and hence, the amount of protein adsorbed on the membrane was determined. Adsorption kinetics of each protein are presented in Fig. 12. HSA and TRF reached its maximum adsorption in clinical module in 8 min, while FB reached the maximum adsorption faster at only 5 min.

Based on adsorption ratio, it also is possible to track the change in composition of adsorbed proteins in comparison with the protein composition in the initial solution. The most adsorbed protein by its ratio was TRF (about 47%) in 8 min, nevertheless, FB approximately achieved the same adsorption ratio in only 5 min, as shown in Fig. 12. Then adsorbed FB was replaced with TRF and HSA, resulting in further decreasing of FB adsorption ratio. As presented in Fig. 12, HSA had initially the least adsorption ratio, which was about 20%.

**Figure 13:**
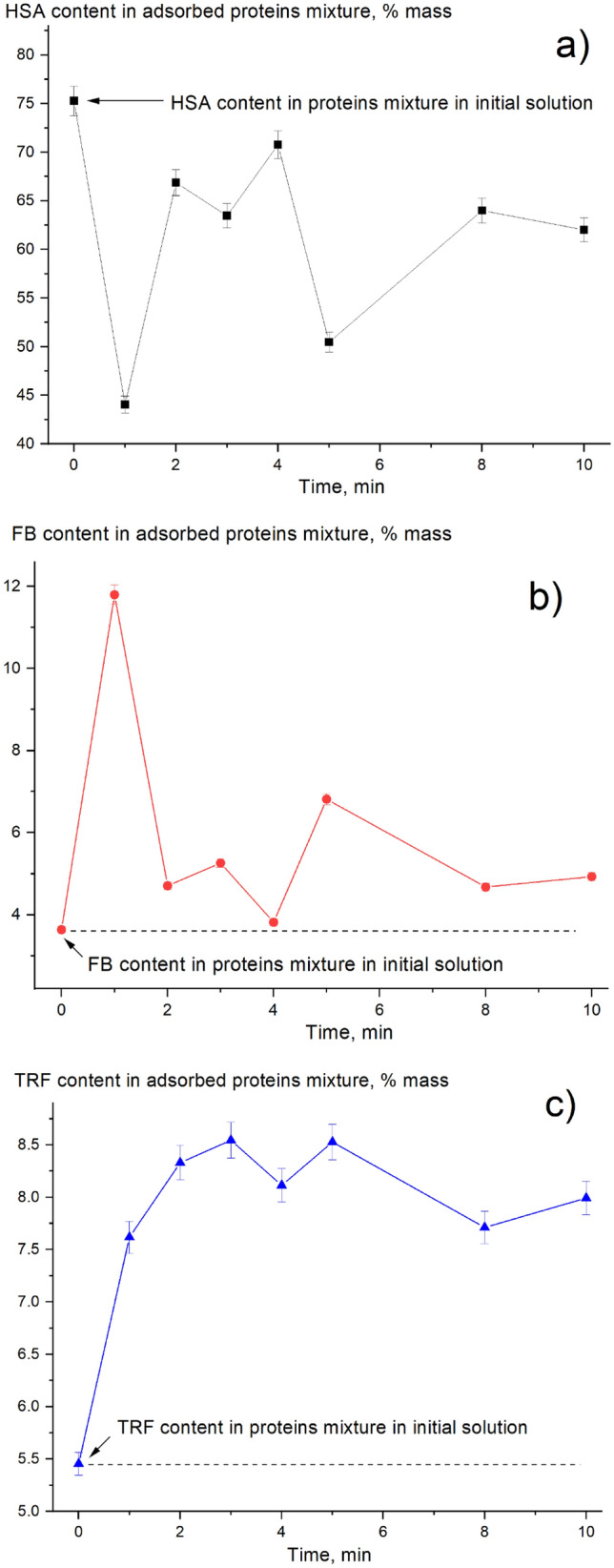
Change in the protein content in the adsorbed protein mixture with time: (**a**) HSA; (**b**) FB; (**c**) TRF.

Furthermore, each protein adsorption ratio in the mixture at different adsorption times was compared to the initial protein content in proteins mixture in the initial solution, as presented in Fig. 13. HSA content significantly decreased from 75% (content of HSA among proteins in initial solution) to 43% in the first minute, as shown in Fig. 13a. On the other hand, FB (see Fig. 13b) and TRF (see Fig. 13c) content increased. Noticeably, a significant change in the protein composition was observed in 1 min of adsorption. FB content increased from 4% (content of FB among proteins in initial solution) to 12%, in the first minute. Furthermore, TRF content increased from 5.5% to 7.5%, in the first minute. This increase in FB and TRF content was compensated by a decrease in the HSA content. Then, part of the adsorbed FB was replaced with HSA and TRF, resulting in the FB content decreasing from 12 to 5%, in the second minute. The second peak of the FB content appeared at 5 min when there was a corresponding drop in the HSA adsorption ratio (see Figs. 12 and 13a,b). It is worth mentioning that protein adsorption contributes to protein depletion ratio from blood stream. Protein losses through dialysis are not negligible and can be as high as 4–8 g/day among both peritoneal and HD patients^38^. In addition, several studies report losses of approximately 1 to 2 g of protein into dialysate with conventional hemodialyzers, but may be higher with high-flux dialyzers^39^.

## Conclusion

The adsorption of three main human blood proteins—human serum albumin (HSA), fibrinogen (FB) and transferrin (TRF), on PES membranes was studied. FB adsorption dominated over other proteins, resulting in a significant lower HSA content in adsorbed layers compared with its content in the initial solution. In addition, the percentage of HSA adsorption onto the HD membrane dropped approximately 10 times when HSA was adsorbed in competition with other proteins, compared to the adsorption from the single HSA solution. Results showed that when proteins were adsorbed from their mixture, FB content (among proteins) in the adsorbed layer increased from 4% mass (content in the initial solution) to 18% mass and 12%, in case of in situ quantitative and in vitro analysis, respectively. The TRF content in the initial solution and the adsorbed layer remained almost the same during adsorption. Though, there was still a distribution of each protein content across the membrane thickness. Moreover, SR-µCT has revealed that FB, adsorbed from a protein mixture, was located predominately in the middle of the membrane, whereas the peak of the distribution shifted to the membrane bottom layers when adsorption from the FB single solution took place. In addition, HSA, FB and TRF adsorption behavior were similar in both *in-situ* small scale and clinical dialyzer with the PES membrane.

## Supplementary Information

Supplementary Information.

## Data Availability

The raw/processed data obtained at the Canadian Light Source (CLS), required to reproduce these findings of this study are available from the corresponding author (Amira Abdelrasoul) on reasonable request.
