# Supplementary material for: In-situ synchrotron quantitative analysis of competitive adsorption tendency of human serum proteins on polyether sulfone clinical hemodialysis membrane
Source: Sci Rep. 2023 Jan 30;13:1692. doi: 10.1038/s41598-023-27596-2 (PMC9886930; doi:10.1038/s41598-023-27596-2)
Supplement: Supplementary file 1 — Supplementary Information. [file 41598_2023_27596_MOESM1_ESM.docx]

***In-situ* Synchrotron Quantitative Analysis of Competitive Adsorption Tendency of Human Serum Proteins on Polyether sulfone Clinical Hemodialysis Membrane**

**Amira Abdelrasoul^1,2*^, Ning Zhu^3^, Huu Doan^4^, Ahmed Shoker^5,6^**

*^1^Department of Chemical and Biological Engineering, University of Saskatchewan, 57 Campus Drive, Saskatoon, Saskatchewan, S7N 5A9, Canada.*

*^2^ Division of Biomedical Engineering, University of Saskatchewan, 57 Campus Drive, Saskatoon, Saskatchewan, S7N 5A9, Canada.*

*^3^ Canadian Light Source, 44 Innovation Blvd, Saskatoon, Saskatchewan, S7N 2V3, Canada*

*^4^Department of Chemical Engineering, Toronto Metropolitan University, 350 Victoria St., Toronto, ON M5B 2K3, Canada*

*^5^ Nephrology Division, College of Medicine, University of Saskatchewan, 107 Wiggins Rd, Saskatoon, SK S7N 5E5*

*^6^ Saskatchewan Transplant Program, St. Paul's Hospital, 1702 20th Street West Saskatoon Saskatchewan S7M 0Z9 Canada*

****Corresponding Author: amira.abdelrasoul@usask.ca, Tel: (306) 966 2946, Fax: (306) 966 4777***

**Supplementary Materials**

**Figure S.1.** UV calibration spectra of (a) FB; (b) HSA; (c) HSA (constant) + FB (variation); and (d) HSA (constant) + FB (constant) + TRF (variation)
